# Strategies for the prevention of perinatal hepatitis B transmission in a marginalized population on the Thailand-Myanmar border: a cost-effectiveness analysis

**DOI:** 10.1186/s12879-017-2660-x

**Published:** 2017-08-09

**Authors:** Angela Devine, Rebecca Harvey, Aung Myat Min, Mary Ellen T. Gilder, Moo Koh Paw, Joy Kang, Isabella Watts, Borimas Hanboonkunupakarn, François Nosten, Rose McGready

**Affiliations:** 1 Centre for Tropical Medicine and Global Health, Nuffield Department of Clinical Medicine, University of Oxford, Oxford, UK; 2 Mahidol-Oxford Tropical Medicine Research Unit, Mahidol University, Bangkok, Thailand; 3 Shoklo Malaria Research Unit, Mahidol-Oxford Tropical Medicine Research Unit, Mahidol University, Bangkok, Thailand; 4 Department of Clinical Tropical Medicine, Faculty of Tropical Medicine, Mahidol University, Bangkok, Thailand

**Keywords:** Hepatitis B, Vaccination, Immunoglobulin, Resource limited settings, Migrants, Refugees

## Abstract

**Background:**

Data on the cost effectiveness of hepatitis B virus (HBV) screening and vaccination strategies for prevention of vertical transmission of HBV in resource limited settings is sparse.

**Methods:**

A decision tree model of HBV prevention strategies utilised data from a cohort of 7071 pregnant women on the Thailand-Myanmar border using a provider perspective. All options included universal HBV vaccination for newborns in three strategies: (1) universal vaccination alone; (2) universal vaccination with screening of women during antenatal visits with rapid diagnostic test (RDT) plus HBV immune globulin (HBIG) administration to newborns of HBV surface antigen positive women; and (3) universal vaccination with screening of women during antenatal visits plus HBIG administration to newborns of women testing HBV e antigen positive by confirmatory test. At the time of the study, the HBIG after confirmatory test strategy was used. The costs in United States Dollars (US$), infections averted and incremental cost effectiveness ratios (ICERs) were calculated and sensitivity analyses were conducted. A willingness to pay threshold of US$1200 was used.

**Results:**

The universal HBV vaccination was the least costly option at US$4.33 per woman attending the clinic. The HBIG after (RDT) strategy had an ICER of US$716.78 per infection averted. The HBIG after confirmatory test strategy was not cost-effective due to extended dominance. The one-way sensitivity analysis showed that while the transmission parameters and cost of HBIG had the biggest impact on outcomes, the HBIG after confirmatory test only became a cost-effective option when a low test cost was used or a high HBIG cost was used. The probabilistic sensitivity analysis showed that HBIG after RDT had an 87% likelihood of being cost-effective as compared to vaccination only at a willingness to pay threshold of US$1200.

**Conclusions:**

HBIG following confirmatory test is not a cost-effective strategy for preventing vertical transmission of HBV in the Thailand-Myanmar border population. By switching to HBIG following rapid diagnostic test, perinatal infections will be reduced by nearly one third. This strategy may be applicable to similar settings for marginalized populations where the confirmatory test is not logistically possible.

**Electronic supplementary material:**

The online version of this article (doi:10.1186/s12879-017-2660-x) contains supplementary material, which is available to authorized users.

## Background

An estimated 240 million individuals are chronically infected with hepatitis B virus (HBV) worldwide [1, 2]. An estimated 686,000 people die globally due to complications of hepatitis B, including liver cirrhosis and hepatocellular carcinoma, which cause approximately 90% of deaths [3]. Transmission occurs through unprotected sex and body fluids [4], including the birth process, which leads to the majority of perinatal infections. Individuals who are HBV e-antigen positive (HBeAg+) are at the highest risk of transmitting to others. Of those who acquire the infection perinatally, 65% become chronic carriers of the disease and therefore are at a higher risk of morbidity and mortality as compared to 28% in those born to HBeAg- mothers [5]. An estimated 5–15% of perinatal infections occur in-utero, which vaccination at birth does not prevent [6]; however, an estimated 90% of perinatal infections could be averted through infant HBV vaccination alongside administration of HBV immunoglobulin (HBIG) to the infant [7–12]. Due to problems of production, storage (cold chain) and cost, most low resource settings do not have access to HBIG. The reduction of mother to child transmission is a core intervention in the World Health Organisation (WHO) global health sector strategy on viral hepatitis, which proposes expansion of coverage from current its level of 38% to 90% by 2030 [13].

The HBV vaccine is the only routine childhood vaccine where prompt administration after birth (within 24 h) is required to prevent transmission [14–16]. A modelling study [17] found that providing the first dose of HBV vaccine at birth could prevent an additional 16% of deaths from the disease, and a more recent study estimated that 210 million chronic infections had been prevented by 2015 through the vaccination of infants and neonates [18]. Universal HBV vaccination with a three to four dose schedule has a protective efficacy of 70–85% [19], which increases up to 95% when administered with HBIG [20]. HBV vaccination has been shown to be cost effective [21–24], and nearly all countries have adopted the WHO recommendation of universal HBV vaccination without screening. In high resource settings, universal vaccination alongside antenatal maternal screening with HBIG administered in hospital to newborns of those who screen HBV surface antigen positive (HBsAg+) is considered to be a cost-effective strategy [7, 25, 26]. These screening strategies are often not taken up by low income countries due to the high costs [1]; yet it is often these low income countries that carry higher burdens of the disease. A lack of information exists on the cost-effectiveness of this strategy in resource limited settings, where late antenatal care is more common and the proportion of births at home is higher.

The Asia-Pacific region disproportionately shares the burden of HBV, with 75% of chronic HBV carriers in the world found in Asia [27]. Vaccine programmes in Lao PDR, Vietnam and Cambodia have successfully lowered the prevalence of chronic HBV carriage to less than 2% by 2012 [28]. Myanmar, however, has continued to have areas of high HBV prevalence, especially in rural and border regions of Myanmar where a prevalence of 8.3% was recently reported [29]. This is widely attributed to the high percentage HBeAg + women of reproductive age in this region.

The aim of this study was to evaluate the cost-effectiveness of options for the prevention of the perinatal HBV transmission in a marginalized (refugee and migrant) population on the Thailand-Myanmar border.

## Methods

### Study site

The Shoklo Malaria Research Unit (SMRU) was established in 1986 in response to the burden of cases and deaths in refugee camps due to malaria and other tropical diseases. Research is conducted alongside humanitarian healthcare work for marginalized populations including Myanmar migrants and refugees on the north western border of Thailand. Thailand and Myanmar are separated by the Moei River, which can be difficult for migrants to cross when trying to access care [30]. On the Myanmar side of the border healthcare is uncoordinated and, in some areas, non-existent or the expected fee for service is insurmountable. While Thailand’s public health systems are strong, options for care for Myanmar women on the Thai side of the border are limited due to socio-economic, language, security and access barriers [31]. HBIG is not provided to non-Thai mothers unless payment can be guaranteed.

SMRU is comprised of three main clinics, one in Mae La refugee camp, and two at migrant sites, Mawker Thai and Wang Pha. Refugee camps were established in the area in 1986 as ethnic Karen from Myanmar fled armed conflict. Mae La refugee camp grew significantly in 1995–6 after additional conflict led to the merger of Shoklo, Bono Klo, Mae La and several other smaller camps. The population at the time of this analysis included an estimated 45,000 refugees [32]. Approximately 5–10% of those who attend the clinic at Mae La refugee camp are migrants or Karen minorities from surrounding villages [31]. The migrant sites, Wang Pha and Mawker Thai established birthing facilities in 2007 and 2010 and provide free services predominantly to agricultural migrants from Myanmar in rural areas 30 km north and 60 km south of Mae Sot, Tak Province, Thailand. Births in SMRU units are described in more detail in White et al. 2016 [33].

Birth in the area has traditionally been at home, which precludes timely HBV vaccination. Significant encouragement by clinic staff and free service provision has reduced home birth to 10–15% [34]. Women who deliver at the clinic are cared for by locally trained skilled birth attendants who can administer vaccinations and will refer women for caesarean sections at Thailand public hospitals if required [32, 34]. Care at the clinic is free and basic public health programmes including routine vaccinations are supported in part by external agencies, including the Thailand Department of Public Health. Treatment for liver cirrhosis, hepatocellular carcinoma and other conditions caused by HBV are unaffordable; and these conditions often result in premature death.

### Strategies for the prevention of HBV transmission

Three strategies were evaluated with all options including universal HBV vaccination of infants:

1. *Vaccine only:* HBV vaccination provided to all newborns. No maternal screening is involved.
2. *HBIG after RDT*: Screening at the first antenatal visit using a point of care rapid diagnostic test (RDT) (One Step Bioline Hepatitis B Surface Antigen Test Strip, Pacific Biotech). Newborns of mothers who test HBsAg + during antenatal care or at delivery are given HBIG.
3. *HBIG after confirmatory test:* As for *HBIG after RDT* (above) with the additional step of confirmation testing in HBsAg + mothers for HBeAg status. Samples are sent to a local hospital to determine HBeAg status with a confirmatory test (HBeAg electrochemiluminescence immunoassay, Roche Diagnostics, USA). Only the newborns of mothers who tests HBeAg + during an antenatal visit are given HBIG and this process requires first antenatal visit at least seven days before delivery to ensure that results are processed in time.

Table 1 summarizes the strategies. The *HBIG after confirmatory test* strategy was the current practice at SMRU at the time of the study; however, the *vaccine only* strategy was used as the base case option since most resource poor settings can provide this option if the cold-chain can be established. While some women do not attend for antenatal care, they first attend the clinic for delivery, which allows their newborns to be vaccinated within 12 h of birth. The newborns of women who deliver at home and present to the clinics within 24 h can also receive HBV vaccine and HBIG if appropriate. In accordance with the immunization policy for Thailand, it was assumed that all newborns who received the vaccine at birth also received the second and third dose of the vaccine.

**Table 1:** Details of the interventions included in each strategy for the prevention of perinatal hepatitis B transmission

	*Vaccine only*	*HBIG after RDT*	*HBIG after confirmatory test*
Vaccine for all newborns born in clinic	X	X	X
Screening for HBsAg with rapid diagnostic test during antenatal visit		X	X
HBIG for newborns born to all mothers who test HBsAg+		X	
Those who test HBsAg + during antenatal visit are screened for HBeAg			X
HBIG for newborns of mothers who attended antenatal clinic and tested HBeAg+			X

A decision tree model was built in R statistical software [35] for a hypothetical cohort of 5000 women using a health care provider perspective. The model structure was based on a similar study in Taiwan by Chen et al. [7] and adapted to the SMRU setting (see Additional File 1). The effectiveness measure was perinatal infection of HBV, caused through vertical transmission of the disease. The time horizon is from first contact with the SMRU clinic until childbirth. The long-term costs and effects of HBV were not included.

### Model parameters

Table 2 shows all parameters used in the model. Parameters for all variables related to the population and clinic were taken from an anonymised mother and baby prospective cohort of 7071 registered mothers from the Thailand-Myanmar border area who either attended antenatal care at SMRU clinics or presented at the clinics to give birth [36]. The data analysis was conducted in STATA [37]. The prevalence of HBsAg in women seeking antenatal and delivery care at the clinic was determined onsite by RDT, and the confirmatory test was used to determine HBeAg status in those who tested HBsAg+. The data was collected using a standardised maternal and child health record used by trained skilled birth attendants and included all mothers registered at and SMRU clinic due to give birth between 1st January 2013 and 31st December 2014. Women who missed antenatal care and attended only for birth, or attended antenatal care but delivered at home and presented at the clinic on the same day were presumed eligible for HBIG. The probability of presenting at the clinic within 12 h was calculated from those who attended antenatal care but delivered at home and presented their newborn at the clinic for cord care and birth weight measurement, as date of birth and date of weight is recorded routinely.

**Table 2:** Parameter values including range used in sensitivity analysis

Parameter	Base value	Low value	High value	Distribution (Parameters^a^)	Source
Probability HBsAg + for all women attending SMRU clinics	0.07	0.07	0.08	Beta (490.6, 6187.1)	SMRU data with 95% CI
Probability HBeAg carrier if HBsAg+	0.34	0.29	0.39	Beta (130.1, 253.6)	SMRU data with 95% CI
Probability women receive antenatal care (attend clinic at least 7 days before delivery)	0.96	0.96	0.96	Beta (999.9, 41.4)	SMRU data with 95% CI
Probability of clinic delivery after attending antenatal care	0.90	0.90	0.91	Beta (999.9, 107.6)	SMRU data with 95% CI
Probability that newborns birthed at home after attending antenatal care will receive HBV vaccine at the clinic (present within 12 h)	0.15	0.12	0.17	Beta (94.3, 552.5)	SMRU data with 95% CI
Probability of HBV perinatal infection for HBeAg- mothers without vaccination	0.11	0.05	0.31	Beta (1.7, 13.5)	[38, 39, 41, 42] with low from [42] and high from [38]
Probability of HBV perinatal infection for HBeAg + mothers without vaccination	0.84	0.66	1.00	Beta (15.5, 2.95)	[38, 40–42] with low from [41] and high from [39]
Probability of HBV perinatal infection for HBeAg- mothers if given vaccine	0.07	0.00	0.13	Beta (4.1, 57.9)	[7]
Probability of HBV perinatal infection for HBeAg + mothers if given vaccine	0.34	0.21	0.43	Beta (24.3, 47.6)	[7]
Probability of HBV perinatal infection for HBsAg- mothers if given vaccine and HBIG	0.01	0.00	0.03	Beta (1.6, 159.0)	[7]
Probability of HBV perinatal infection for HBeAg + mothers if given vaccine and HBIG	0.13	0.06	0.29	Beta (3.1, 21.7)	[7]
Sensitivity of the RDT for HBsAg	0.98	0.90	1.00	Beta (24.3, 0.5)	[44] with assumed range
Specificity of the RDT for HBsAg	0.97	0.95	0.98	Beta (306.4, 9.9)	[29] with 95% CI
Sensitivity of the confirmatory test for HBeAg	1.00	0.90	1.00	Beta (40.0, 0.0)	[43] with assumed range
Specificity of the confirmatory test for HBeAg	1.00	0.90	1.00	Beta (40.0, 0.0)	[43] with assumed range
*Costs*					
Cost of HBV vaccinations	4.71	2.36	7.07	Gamma (16.1, 0.3)	SMRU records ±50%. Cost of single vaccination at birth plus two doses of HBV diphtheria tetanus and pertussis combined vaccine given at 2 and 6 months
Cost of a RDT for HBsAg	1.17	0.59	1.76	Gamma (16.1, 0.1)	SMRU records ±50%
Cost of a confirmatory test for HBeAg at local hospital	17.85	8.93	26.78	Gamma (16.1, 1.1)	SMRU records ±50%
Cost per dose of HBIG	43.00	21.50	64.50	Gamma (16.1, 2.7)	SMRU records ±50%

All costs are in 2015 United States Dollars. Confidence interval (CI)

^a^Parameters: Beta (alpha, beta), Gamma (shape, scale)

Transmission rates when using vaccine or vaccine with HBIG were taken from a previous economic evaluation [7]. Transmission rates without vaccine were taken from a literature review of studies in Asia [38–42]. The diagnostic accuracy of the confirmatory test was taken from the literature [43] as was the sensitivity of the RDT [44]. The specificity of the RDT was taken from a local study that showed that it was lower than previously reported [29].

Unit costs were taken from the 2015 financial records of the clinics and include those for diagnostic tests, vaccination and HBIG at the clinic. As all women are encouraged to deliver at the clinics, the cost of delivery was not included. All costs are reported in 2015 United States Dollars (USD). Unit costs that were reported as Thai Baht were converted into USD using the average exchange rate for 2015 (1 Thai baht = 0.029 USD) [45].

### Analysis

The total costs and perinatal infections were calculated for each strategy, and the results were plotted on a cost-effectiveness plane. The strategies were then ordered from the least to the most expensive. Any options that were dominated due to averting fewer perinatal infections than the previous less expensive strategy were then removed. The incremental cost effectiveness ratio (ICER) for the non-dominated strategies was calculated using the following formula:

$$ \frac{C_B-{C}_A}{-\left({E}_B-{E}_A\right)} $$

Any options with extended dominance were then removed from the analysis. A willingness to pay threshold of US$1200 was used, which is one gross domestic product (GDP) per capita for Myanmar [46]. A one way sensitivity analysis was conducted to test the impact of each parameter on the ICER and whether each strategy was cost effective. ICER changes greater than 1% of the base case ICER were reported. A probabilistic sensitivity analyses (PSA) was conducted to incorporate the uncertainty of parameter estimates over 10,000 sampling iterations. The sum of squared differences was minimized from the specified ranges to produce the shape values for the beta and gamma distributions and random numbers were generated from these distributions. The PSA produced a mean estimate and 95% credible intervals (CrIs) for the results. The low and high values and distributions used for the sensitivity analyses are shown in Table 2. Since this setting is resource-constrained, a one GDP per capita threshold could still be too high [47, 48], though it is plausible that averting an infection would avert more than one disability-adjusted life-year. Accordingly, the cost-effectiveness acceptability curve produced by the PSA examined the likelihood of interventions being cost-effective at different willingness to pay thresholds.

## Results

**Fig. 1:**
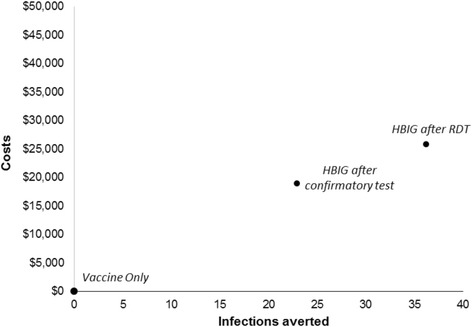
Cost-effectiveness plane with *vaccine only* as the base case comparator for infections averted

Table 3 presents the results for the base case analysis. The cohort costs ranged from US$21,673.15 for the *vaccine only* strategy to US$47,477.10 for the *HBIG after RDT* strategy; these are equivalent to US$4.33 to US$9.50 per woman presenting at the clinic. The *vaccine only* had the lowest effectiveness with 64 perinatal infections in the hypothetical cohort of 5000 newborns. The number of infections was reduced to 41 with the *HBIG after confirmatory test* and 28 with the *HBIG after RDT* strategy. The *HBIG after confirmatory test* strategy were removed due to extended dominance by the *HBIG after RDT* strategy, which had an ICER of US$716.78 per infection averted, which was below the willingness to pay threshold of US$1200. The PSA produced a 95% CrI of US$343.00–2159.90 for the ICER. Figure 1 shows the cost-effectiveness plane.

**Table 3:** Cost-effectiveness results for cohort of 5000 women (costs are in USD)

Strategy	Total costs	Incremental costs	Total infections	Infections averted	ICER^a^
*Vaccine only*	$21,673.15	base case	64	base case	base case
*HBIG after confirmatory test*	$40,553.86	--	41	--	extended dominance
*HBIG after RDT*	$47,477.10	$25,803.95	28	36	$716.78

^a^ICER = Incremental cost-effectiveness ratio

**Fig. 2:**
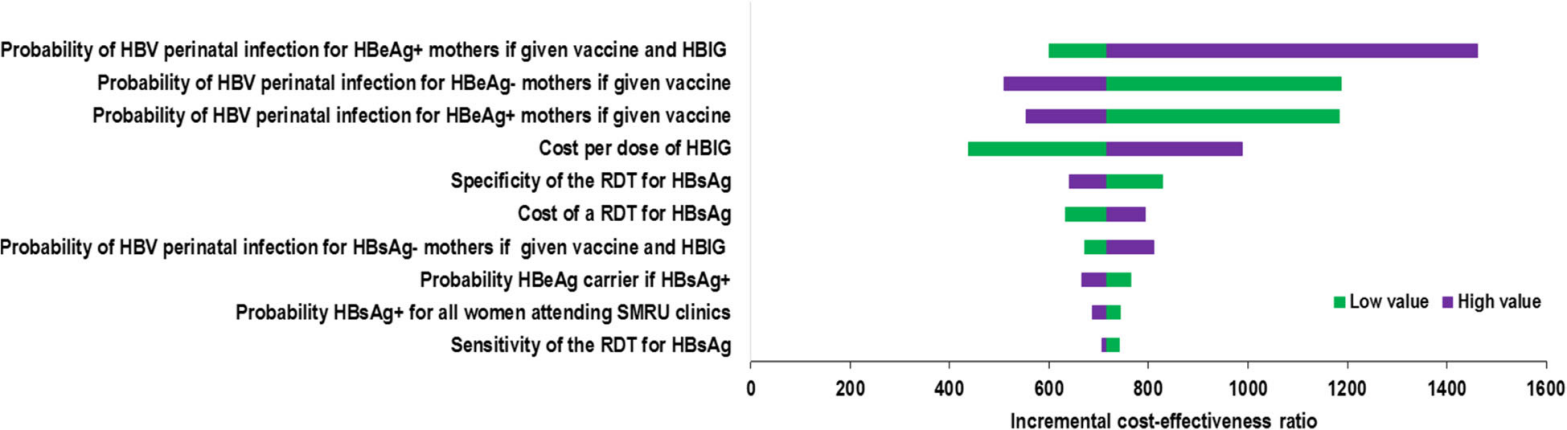
One way sensitivity analysis results of the incremental cost-effectiveness ratio for *HBIG after RDT* strategy compared to *vaccine only*

Figure 2 shows the results of the one way sensitivity analysis on the ICER for *HBIG after RDT* strategy as compared to the *vaccine only* strategy. The three parameters that had the largest impact on the ICER of the *HBIG after RDT* strategy were all related to transmission: the probability of HBV perinatal infection for both HBeAg + and HBeAg- mothers when newborns were given vaccine and the probability of HBV perinatal infection for HBeAg + mothers if newborns were given vaccine and HBIG. A 50% change in the cost of HBIG also caused the ICER to change by nearly 40% in both directions with a low cost causing a reduction in the ICER. When the high value for the probability of HBV perinatal infection for HBeAg + mothers when newborns were given the vaccine and HBIG was raised to 43%, the *HBIG after RDT* strategy had an ICER of US$1462, which was the only time the strategy rose above the threshold of US$1200. For all other parameter values evaluated in the one-way sensitivity analysis, the *HBIG after RDT* strategy remained below US$1200.

**Fig. 3:**
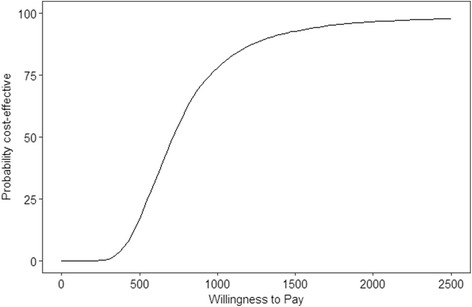
Cost-effectiveness acceptability curve for *HBIG after RDT* as compared to *vaccine only*

The *HBIG after confirmatory test* was only non-dominated for two parameter values. The first was when a low cost for the confirmatory test (US$8.93) was used, which resulted in an ICER of US$636.14 for the *HBIG after confirmatory test* and an ICER of US$846.16 for the *HBIG after RDT strategy*. The second parameter value was the high HBIG cost (US$64.50) was used. This resulted in ICERs of US$925.90 and US$1097.90 for the *HBIG after confirmatory test* and the *HBIG after RDT* strategy, respectively. Figure 3 shows the cost-effectiveness acceptability curve for the comparison between *HBIG after RDT* and *vaccine only*. At a willingness to pay threshold of US$1200 per perinatal infection averted, the *HBIG after RDT* strategy had an 87% likelihood of being cost-effective. If the willingness to pay threshold was lowered to US$600 per infection averted, the likelihood of the *HBIG after RDT* strategy being cost-effective dropped to 32%.

## Discussion

Our results indicate that two cost-effective strategies are available for the prevention of perinatal transmission of HBV in a population on the Thailand-Myanmar border. With an ICER of US$716.78, the *HBIG after RDT* strategy is cost-effective at the one GDP per capita threshold of US$1200 per hepatitis B infection averted, which would result in even greater benefits in terms of disability-adjusted life years averted. In such a resource-constrained setting where a dose of HBIG can be equivalent to one month’s salary for the average family, this intervention may still not be feasible. Assuming that funding is available, a switch from the current practice of *HBIG after confirmatory test* to the *HBIG after RDT* strategy, infections could be reduced by nearly a third for a relatively small increase in the overall programme costs. Importantly, the *HBIG after RDT* strategy was consistently a cost-effective option in the one-way sensitivity analysis. Our results are similar to a study conducted in Taiwan, where maternal screening for HBsAg and HBIG given to all infants of HBV positive mothers along with universal vaccination was also found to be cost effective at all levels of endemicity [7].

Two cost parameters, the low value for the confirmatory test and the high value for the HBIG, had a significant impact on the results of the one-way sensitivity analysis, shifting the *HBIG after confirmatory test* to become a cost-effective strategy. As confirmatory tests require facilities found at a hospital, it is unlikely to drop in price by 50% to the low value of US$8.93 that was used in this analysis. Ideally, a new rapid diagnostic test would have confirmatory capability and could make the *HBIG after confirmatory test* a more viable option, as HBIG costs are also very unlikely to come down in price.

Few economic evaluations of strategies involving HBV screening in low resource settings have been published; these studies mainly focus on universal vaccination, which is consistently cost-effective with the exception of very low endemicity settings [21, 24, 49–52]. In most high resource settings, a low prevalence of HBV has produced mixed cost-effectiveness results [53–55]. Many earlier studies have found that universal maternal screening programmes are not cost effective compared to screening only those who are at high risk; however, more recent studies have found universal antenatal screening for HBV to be cost effective [56, 57]. In almost all high resource settings, HBIG is offered to infants born to known HBsAg + mothers. The costs of HBIG are generally covered by long-sighted policies for HBV control. Recent publications suggest that offering HBIG only to those who are willing to pay for it would result in zero uptake in marginalized populations existing on subsistence daily wages due to the high cost [58].

There is evidence from previous settings of high endemicity that HBV transmission can be reduced and elimination of the disease could be possible [59]. The use of HBIG could accelerate this process and this study has shown it is a cost effective strategy to use in a low resource setting. As settings achieve lower endemicity, the cost-effectiveness of interventions to reduce mother to child transmission of HBV will decrease [7, 50], which may make it even less viable to continue with the *HBIG after RDT* strategy when this money could be directed towards other healthcare needs.

The RDT in use at the site carries a false positive rate of 3.1% (95%CI 1.7–5.4) [29], which raises the possibility of a child receiving HBIG when they do not need it if the *HBIG after RDT* strategy were adopted. Common mild adverse effects of HBIG include pyrexia, malaise, drowsiness and urticaria with rare reports of serious adverse effects including anaphylaxis [60]. The *HBIG after confirmatory test* option eliminates this problem, but we were unable to find estimates in the literature for the rate of serious events of HBIG administration in neonates without HBV, making it difficult to directly include in the model. In addition, when the specificity of the RDT was reduced to 95%, which is the low end of the CI recently reported by Banks et al. for this population [29], the ICER rose from US$716.78 to US$828.97, decreasing the cost-effectiveness of the *HBIG after RDT* strategy. Poor diagnostic accuracy has been reported in the literature so finding a locally-distributed test with better specificity may be advantageous [61]. RDT tests for HBV have significantly raised the safety of blood for transfusion in resource limited settings when used to exclude potentially positive cases from donating blood [61]. Pragmatically the same RDT used to screen blood donors was used to screen pregnant women at the point of care in this setting. While tests with better diagnostic accuracy are being developed, their feasibility in limited-resource settings must be considered [61]. A more specific RDT would increase the cost-effectiveness of the *HBIG after RDT* strategy, but the increased cost associated with such an RDT may offset this or cause it to be less cost-effective than reported here.

Our study has several limitations. Firstly, long-term effects including morbidity, mortality and costs and effects of HBV infection are not evaluated in our analysis. Also, not all of those who are infected with HBV will suffer long term damage from the disease. Of those infected at birth, 15–25% will acquire cirrhosis of the liver or develop hepatic carcinoma [4, 62]. In clinics such as SMRU, and most limited-resource settings where HBV and associated diseases cannot be treated due to resource constraints, the health gains associated with more thorough neonatal intervention are considerable [63].

Another limitation is the exclusion of data on multiple births, stillbirths, miscarriages and women who delivered at other clinics or hospitals. Approximately 1% of women in this population have multiple births. These could impact the model results; however, due to the robustness of the model in relation to transmission rate changes, it is unlikely to alter the cost effectiveness of any of the strategies. Other important data limitations relate to the uncertainties surrounding the parameters of the model. In particular, a great deal of uncertainty surrounds the transmission probabilities, especially for those who do not receive vaccination or HBIG. For transmission rates for HBsAg carriers and HBeAg carriers who did not receive a vaccine or vaccine with HBIG, the model was robust to variation in the sensitivity analysis. The transmission rates for HBsAg carriers and HBeAg carriers who were vaccinated with or without HBIG, however, had a sizeable impact on the ICER.

Finally, the HBV transmission rates after vaccination and after vaccination and HBIG were based on studies where participants had received all three vaccines as this is the recommendation of the Thai government. Yet in this population, as in most low resource settings, low rates of full vaccination coverage exist. While in Mae La refugee camp the full course coverage was as high as 98%, it may be much lower in migrant settings where access or migration due to work result in reduced completion rates [64]. The effect of the vaccination and HBIG on vertical transmission of HBV will most likely be provided by the dose given at birth and therefore poor uptake of the second and third vaccine would have a low impact on the results of this study [17]. Newer treatment modalities, such as the use of tenofovir to reduce vertical transmission of HBV in resource limited settings, should be considered due to their improved efficacy and potential cost benefits [65]. Tenofovir would be ideal in middle to high HBV endemic settings with high rates of homebirth. The high price of vaccines [66] alongside country-wide HBIG shortages experienced in this setting in 2016 make exploring the option of Tenofovir even more attractive.

## Conclusions

This study presents the first economic evaluation of strategies for the prevention of vertical transmission in a marginalized population of HBV on the Thailand-Myanmar border population. The results demonstrate that the current clinical strategy of *HBIG after confirmatory test* was not cost effective when compared to *HBIG after RDT*. Barriers to implementing *HBIG after RDT* strategy in limited-resource settings remain including: quality of the HBV RDT (risking administration of HBIG to infants without risk of HBV), HBIG supply problems, and home births. This study adds to the very limited body of literature on the cost effectiveness of HBV prevention strategies for vertical transmission in low resource settings. The use of *HBIG after RDT* could be considered in low resource settings, particularly those with high HBV prevalence; however, the need for more cost-effective options for low resource settings is urgent.

## Additional file

Additional file 1: Model structure. Decision tree diagrams for hepatitis B prevetion in newborns: (A) *Vaccine only* (B) *HBIG after RDT* (C) *HBIG after confirmatory test*. (PDF 99 kb)

## Abbreviations

HBeAg +: Hepatitis B e-antigen positive
HBIG: Hepatitis B immunoglobulin
HBsAg +: Hepatitis B surface antigen positive
HBV: Hepatitis B virus
ICER: Incremental cost-effectiveness ratio
RDT: Rapid diagnostic test
SMRU: Shoklo Malaria Research Unit
WHO: World Health Organisation
