# Supplementary figures and images for: Strategies for the prevention of perinatal hepatitis B transmission in a marginalized population on the Thailand-Myanmar border: a cost-effectiveness analysis
Source: BMC Infect Dis. 2017 Aug 9;17:552. doi: 10.1186/s12879-017-2660-x (PMC5550954; doi:10.1186/s12879-017-2660-x)

A

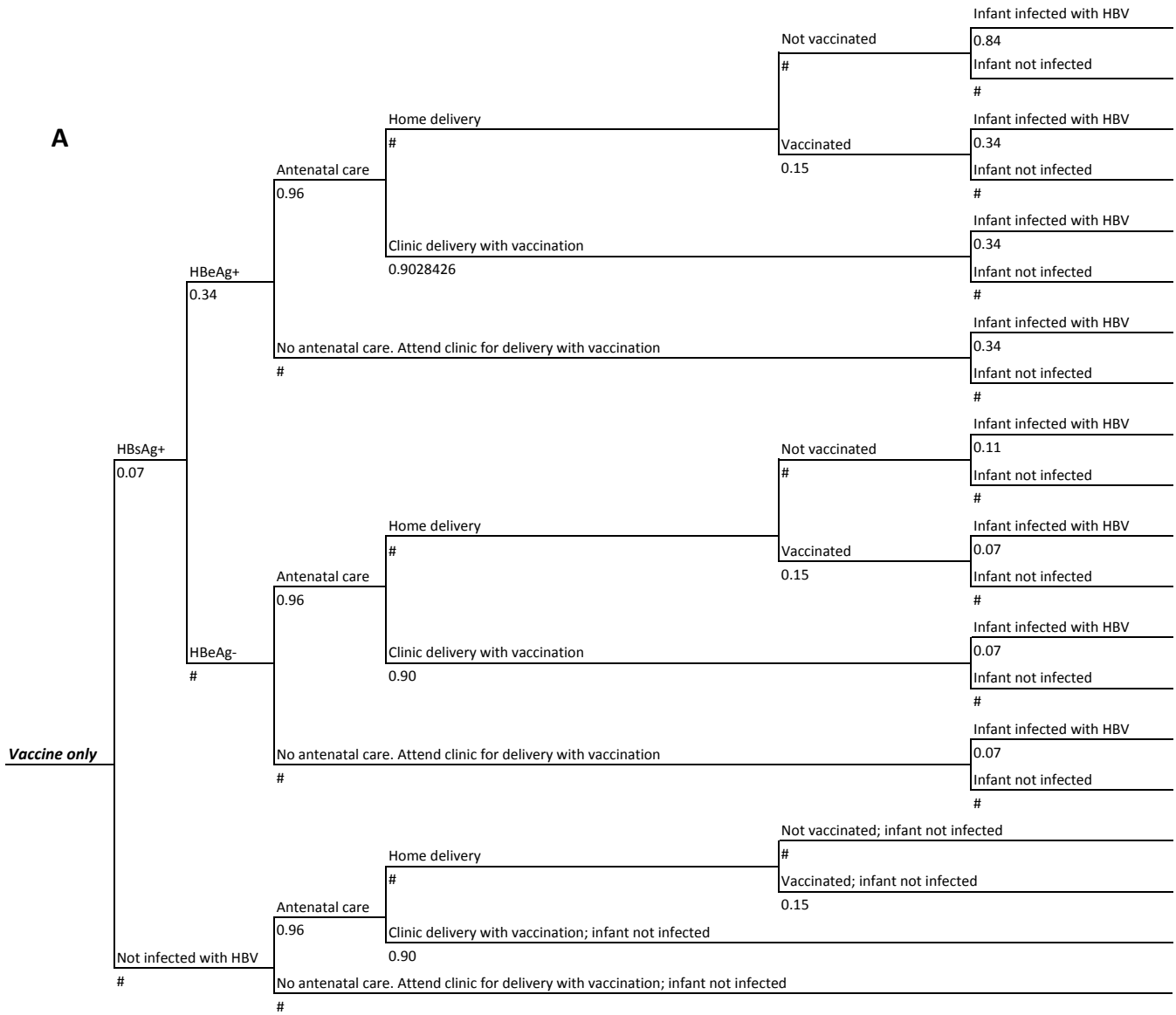

B

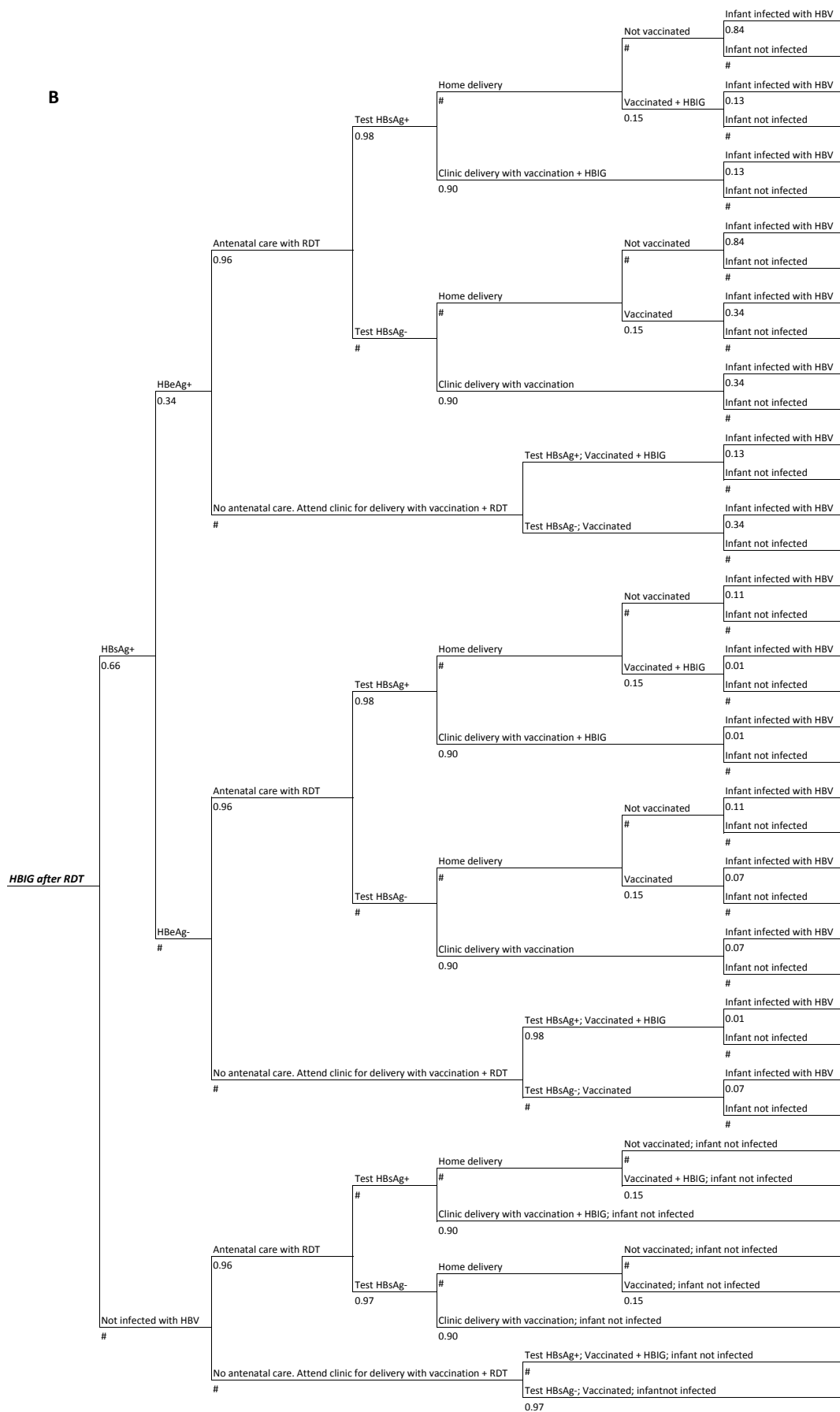

C

*HBIG after  
confirmatory test*

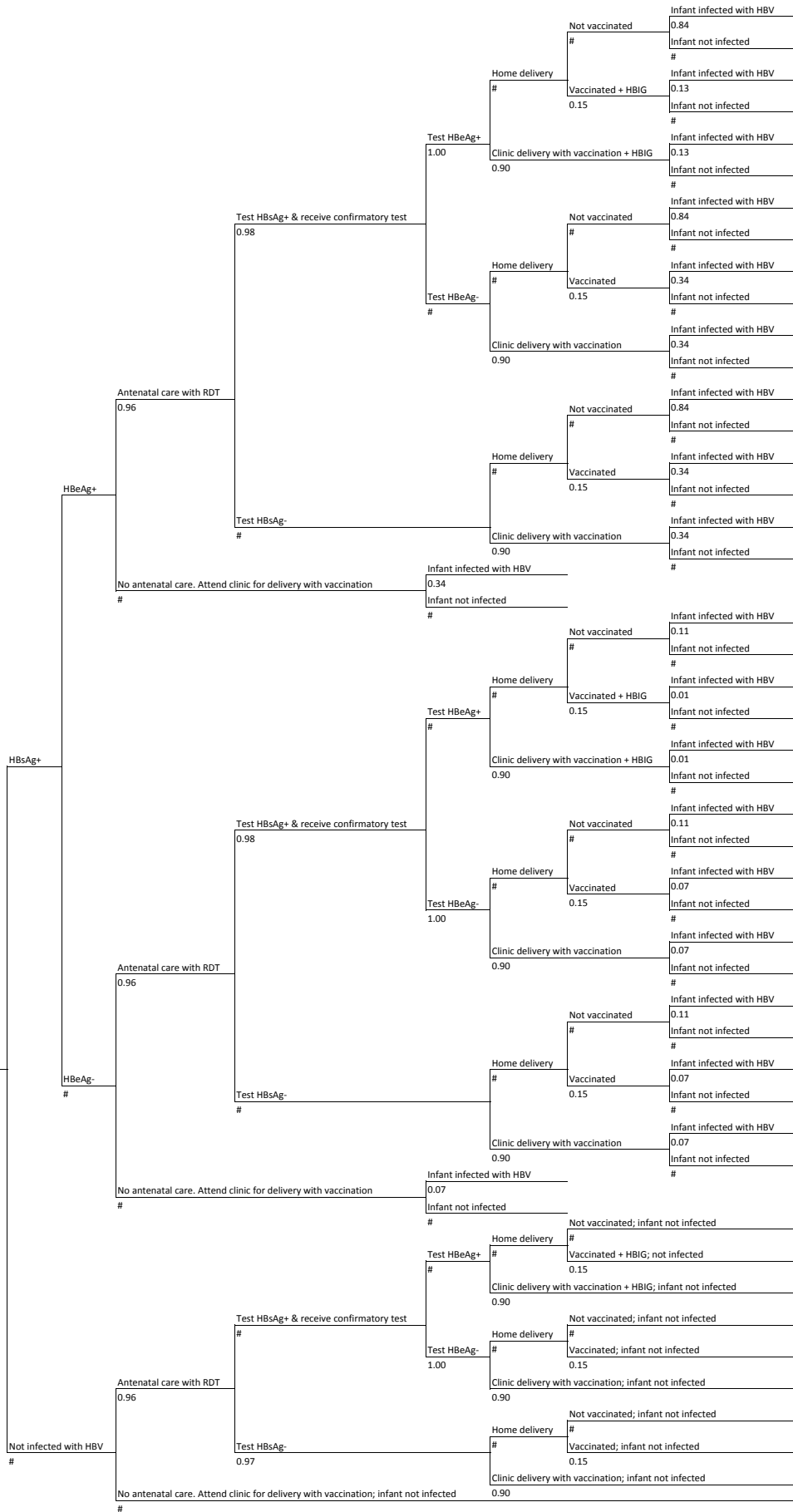

Supplement: Additional file 1: — Model structure. Decision tree diagrams for hepatitis B prevetion in newborns: (A) Vaccine only (B) HBIG after RDT (C) HBIG after confirmatory test. (PDF 99 kb) [file 12879_2017_2660_MOESM1_ESM.pdf]
